# Chloramidine/Bisindolylmaleimide-I-Mediated Inhibition of Exosome and Microvesicle Release and Enhanced Efficacy of Cancer Chemotherapy

**DOI:** 10.3390/ijms18051007

**Published:** 2017-05-09

**Authors:** Uchini S. Kosgodage, Rita P. Trindade, Paul R. Thompson, Jameel M. Inal, Sigrun Lange

**Affiliations:** 1 Cellular and Molecular Immunology Research Centre, School of Human Sciences, London Metropolitan University, 166-220 Holloway Road, London N7 8DB, UK; uck0002@my.londonmet.ac.uk; 2 University College London School of Pharmacy, 29-39 Brunswick Square, London WC1N 1AX, UK; ana.trindade.14@ucl.ac.uk; 3 Department of Biochemistry and Molecular Pharmacology, University of Massachusetts Medical School, Worcester, MA 01655, USA; Paul.Thompson@umassmed.edu; 4 Department of Biomedical Sciences, University of Westminster, 115, New Cavendish Street, London W1W 6UW, UK

**Keywords:** microvesicles, exosomes, drug retention, prostate cancer, peptidylarginine deiminase, chloramidine, bisindolylmaleimide-I, 5-fluorouracil, multidrug resistance

## Abstract

Microvesicle (MV) release from tumour cells influences drug retention, contributing to cancer drug resistance. Strategically regulating MV release may increase drug retention within cancer cells and allow for lower doses of chemotherapeutic drugs. The contribution of exosomes to drug retention still remains unknown. Potential exosome and MV (EMV) biogenesis inhibitors, tested on human prostate cancer (PC3) cells for their capacity to inhibit EMV release, were also tested on PC3 and MCF-7 (breast cancer) cells for improving chemotherapy. Agents inhibiting EMV release most significantly, whilst maintaining cell viability, were chloramidine (Cl-amidine; 50 µM) and bisindolylmaleimide-I (10 µM). Apoptosis mediated by the chemotherapy drug 5-fluorouracil (5-FU) was significantly enhanced in PC3 cells in the presence of both these EMV inhibitors, resulting in a 62% (Cl-amidine + 5-FU) and 59% (bisindolylmaleimide-I + 5-FU) decrease in numbers of viable PC3 cells compared to 5-FU alone after 24 h. For MCF-7 cells, there were similar increased reductions of viable cells compared to 5-FU treatment alone ranging from 67% (Cl-amidine + 5-FU) to 58% (bisindolylmaleimide-I + 5-FU). Using combinatory treatment, the two EMV inhibitors further reduced the number of viable cancer cells tested. Neither inhibitor affected cell viability. Combining selected EMV inhibitors may pose as a novel strategy to enhance the efficacy of chemotherapeutic drug-mediated apoptosis.

## 1. Introduction

Exosomes and microvesicles (MVs)—also referred to as EMVs—are released from all cell types and are key mediators of intercellular communication. EMVs comprise exosomes (40–150 nm in diameter), released through exocytosis, and MVs (150–1000 nm in diameter) that bud off directly from the plasma membrane [1,2]. These vesicles carry lipids and proteins characteristic of their parental cells and affect the physiology of recipient cells by inducing intracellular signalling following receptor binding or by delivering new receptors, enzymes, cytokines, or even genetic material, including miRNA [3].

Multidrug resistance (MDR) describes the capacity of cancer cells to resist chemotherapy. Due to our incomplete understanding, MDR has become a major challenge to effective treatment [4]. It is known that cancer cells use ATP-dependent and an independent efflux of various cancer drugs [5]. Whilst many of the MDR proteins belong to the ATP-binding cassette (ABC) transporter family [6,7,8], including MDR protein (MRP), Breast Cancer Resistance Protein (BCRP), and P-glycoprotein (P-gp), some, such as lung-resistance related protein (LRP), are not ABC transporters and yet are still involved in drug uptake [9].

EMVs can also transfer P-gp, which is involved in MDR [10], and as any change in EMV-mediated communication is likely to affect MDR [11,12], regulation of EMVs provides a putative new strategy to prevent MDR and improve treatment.

In cancer chemotherapy, it is essential that the drug be retained within the target cell to improve efficacy. Microvesicle release has been shown to affect drug retention within cancer cells. This is because chemotherapeutic drugs stimulate cells to release MVs, which we and others have shown carry these drugs within them [12,13,14,15,16,17,18,19,20,21]. Inhibition of MV release has been shown [12,13,14,15,16,17] to increase retention within cells and to sensitize them to chemotherapy. The aim of this study was to measure the capacity of various candidate EMV inhibitors to inhibit EMV release from prostate cancer cells (PC3). Following previous work in our lab, in which MV inhibitors calpeptin and chloramidine (Cl-amidine) rendered PC3 cells more sensitive to chemotherapy, in the same vein we tried some of the new inhibitors as well as combinations of these inhibitors (some of which also inhibited exosome release) in experiments using 5-fluorouracil (5-FU).

## 2. Results

### 2.1. Cl-amidine, Bisindolylmaleimide-I, and Imipramine Effectively Inhibit Exosome and Microvesicle (EMV) Release from PC3 Cells

**Figure 1:**
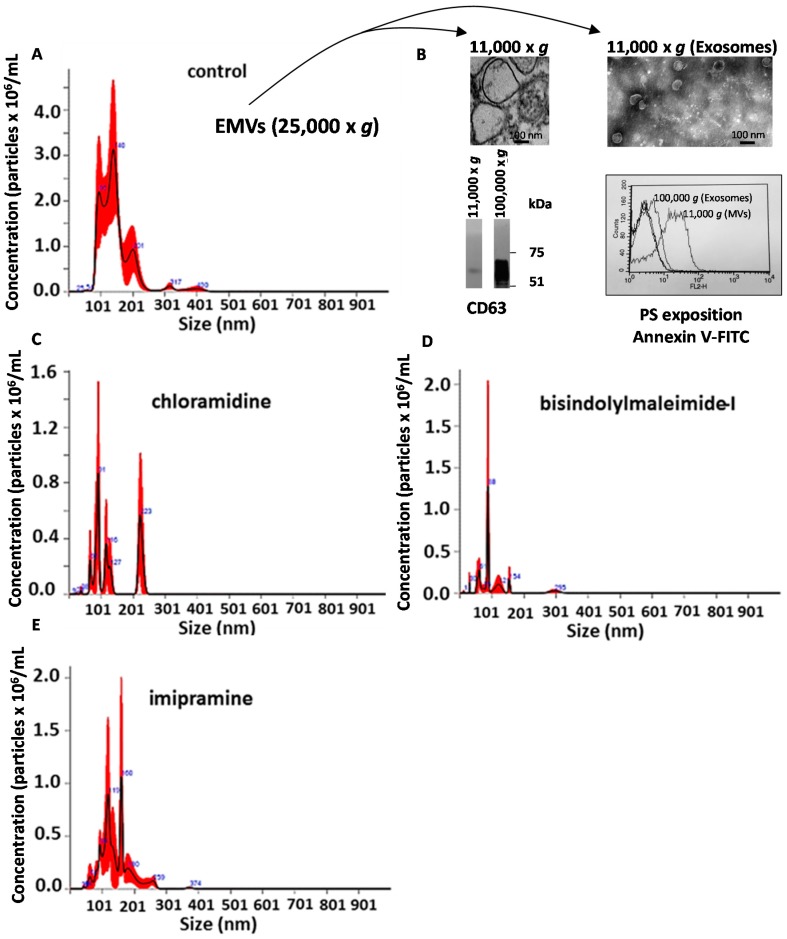
Nanoparticle tracking analysis (NTA) of exosomes and microvesicles (EMVs) released from PC3 cells in the presence of a range of EMV inhibitors. Plots presenting NTA analysis show the concentration of vesicles (0–900 nm in diameter) released from PC3 cells in the absence of any EMV inhibitors (**A**); In (**B**) the EMVs are shown to comprise exosomes and microvesicles (MVs) by electron microscopy, by Western blotting (for CD63 expression), and by the degree of phosphatidylserine (PS) exposition. NTA analysis for released EMVs from PC3 cells are presented in the presence of Cl-amidine (**C**); bisindolylmaleimide-I (**D**); and imipramine (**E**). Vesicles outside the size range of 0–900 nm were excluded to avoid including larger vesicles such as MV aggregates or apoptotic bodies. The experiment was repeated three times in total (error bars ± SEM, indicated in red).

**Figure 2:**
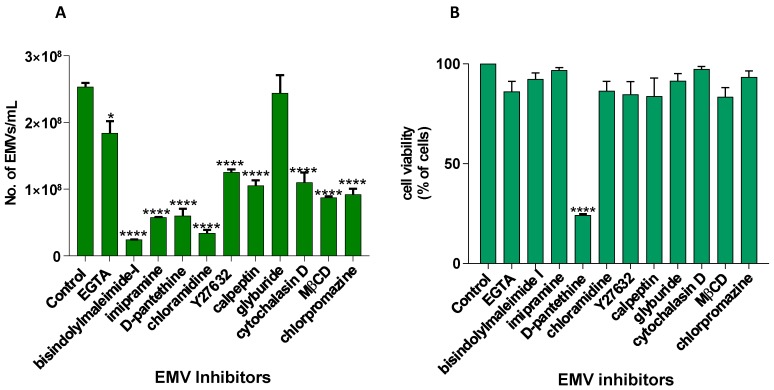
Pharmacological inhibition of EMV release from PC3 cells is highest with Cl-amidine, bisindolylmaleimide-I, and imipramine. (**A**) Using NTA, the most significant inhibition of EMV release from PC3 cells after 24 h was observed in the presence of Cl-amidine, bisindolylmaleimide-I, and imipramine. After 24 h, none of the inhibitors caused any significant reduction in cell viability, (**B**) except for d-pantethine. The experiments were repeated three times, and the data presented are mean ± SEM of the results. (* *p* ≤ 0.05; **** *p* ≤ 0.0001).

EMVs were collected from cells after 24 h and analysed by nanoparticle tracking analysis NTA (Figure 1A). The vesicles collected at 25,000× *g* were confirmed (Figure 1B) to comprise EMVs by separate isolation of MVs (centrifugation at 10,000× *g*) and of exosomes (100,000× *g*) as determined by electron microscopy (MVs ≥ 150 nm; exosomes ≤150 nm), by the expression of CD63 (negligible in MVs, strong in exosomes), and by a higher PS exposition in MVs compared to exosomes. Cells were then treated with a range of pharmacological agents and EMV counts determined by NTA analysis (Figure 1C–E and Figure 2A). A Guava ViaCount assay showed that the viability of cells was maintained throughout, except with d-pantethine, which reduced viability by almost 80% (Figure 2B). Cl-amidine (50 µM) and bisindolylmaleimide-I (10 µM) gave the maximum inhibition of EMV release resulting in 87% and 90% inhibition, respectively. Imipramine was also found to be a potent EMV inhibitor, causing a 77% reduction in EMV count (Figure 2A). All the remaining reagents tested, except for glyburide, also resulted in a significant inhibition. This suggests that these inhibitors are able to inhibit a range of different pathways of EMV biogenesis and might therefore be used individually or in combination to develop more potent EMV inhibiting strategies.

**Figure 3:**
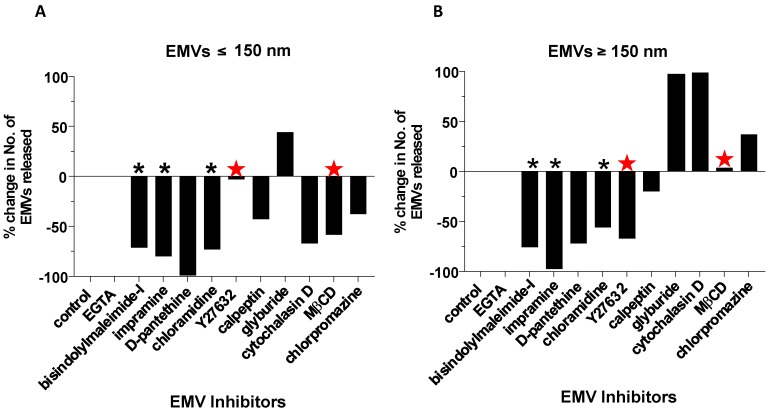
Size exclusion analysis of the NTA data, considering vesicles ≤150 nm (**A**) or ≥150 nm (**B**), indicates percentage changes in number of exosome-sized vesicles (**A**) versus MV-sized vesicles (**B**) respectively. MβCD, which only reduced exosome-sized vesicles (≤150 nm), and Y27632, which only reduced MV-sized vesicles (≥150 nm), are both indicated by red stars. Cl-amidine, bisindolylmaleimide-I, and imipramine, which inhibited both exosome-sized and MV-sized vesicles, are indicated by black asterisks.

Having revealed several reagents that inhibit the total number of EMVs released from PC3 prostate cells, the NTA data was further analysed, based on size exclusion, to verify whether the inhibition detected was equal for typically exosome-sized vesicles (≤150 nm) and for typically MV-sized vesicles (≥150 nm). MβCD, a commonly used endocytosis inhibitor, reduced the number of exosome-sized vesicles compared to the untreated control by 58% and exclusively affected vesicles in this size range, with numbers of MV-sized vesicles barely changing compared to the control (Figure 3A,B, red stars). In contrast, Y27632, which reduced the number of MV-sized vesicles by 67%, only had a minimal effect (3% reduction) on exosome-sized vesicles (Figure 3A,B; red stars). Bisindolylmaleimide-I, Cl-amidine, and imipramine caused significant reductions of both ≤150-nm- and ≥150-nm-sized vesicles (Figure 3A,B; black asterisks). It was noteworthy, therefore, that, according to particle size analysis, the most effective inhibitors of EMV release, bisindolylmaleimide-I, Cl-amidine (and imipramine) reduced numbers of both MVs and exosomes; while this was not the case with Y27632, calpeptin, MβCD, cytochalasin D, and chlorpromazine, which showed a specific tendency to reduce only one vesicle subtype.

### 2.2. Synergistic Effect of Cl-Amidine and Bisindolylmaleimide-I on 5-FU-Mediated Apoptosis of PC3 Cells

Combinations of bisindolylmaleimide-I and Cl-amidine were used to test for a synergistic effect of EMV inhibitors in increasing the efficacy of the anti-cancer drug, 5-fluorouracil (5-FU). PC3 and MCF-7 cells were given 50 µM Cl-amidine and 10 µM bisindolylmaleimide-I separately or in combination while adding 1 µM 5-FU; control cells were treated with 1 µM 5-FU only. After 24 h, the Guava ViaCount cell death assay was performed on the Guava EasyCyte 8HT flow cytometer.

**Figure 4:**
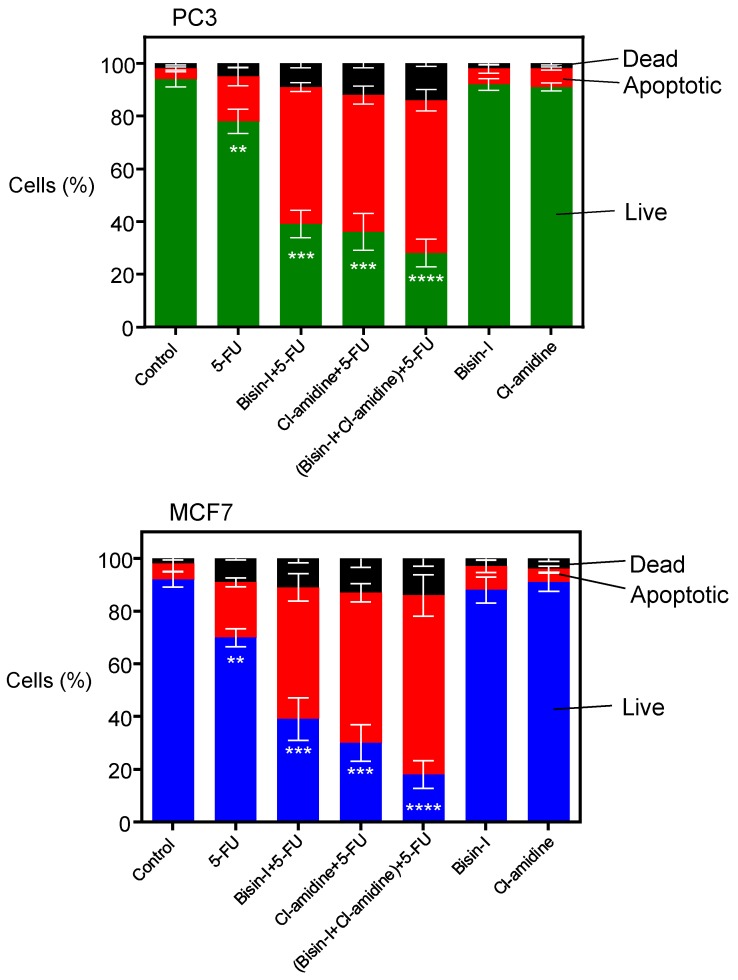
Cl-amidine and bisindolylmaleimide-1-mediated inhibition of EMV release increases the apoptosis of PC3 and MCF-7 cells treated with 5-FU. The Guava Viacount Cell Death Assay shows that PC3 and MCF-7 cells that were given 5-FU together with Cl-amidine, bisindolylmaleimide-I, or with a combination of Cl-amidine and bisindolylmaleimide-I, had significantly reduced levels of cell viability within 24 h compared to PC3 and MCF-7 cells receiving no EMV inhibitors and given only 5-FU. Bisindolylmaleimide-I and Cl-amidine had no significant effect on cell viability on their own. Data presented are the mean ± SEM of three independent experiments performed in triplicate (** *p* ≤ 0.01; *** *p* ≤ 0.001; **** *p* ≤ 0.0001 were considered statistically significant compared to the drug-treated control in the absence of inhibitors).

PC3 and MCF-7 cells given Cl-amidine or bisindolylmaleimide-I alongside 5-FU had a markedly increased level of apoptosis compared to those treated with 5-FU alone (Figure 4). Combinatory treatment with the two EMV inhibitors (50 µM Cl-amidine and 10 µM bisindolylmaleimide-I) induced a still greater level of apoptosis in the presence of 5-FU, compared to cells treated with 5-FU alone (Figure 4). Both bisindolylmaleimide-I and Cl-amidine on their own had negligible effects on cell viability at 24 h (Figure 4).

## 3. Discussion

This study reveals a range of pharmacological agents that can effectively inhibit the release of cellular EMVs, selectively affecting a range of pathways for EMV biosynthesis. All the prospective inhibitors of EMV release tested, bar glyburide, resulted in a clear EMV inhibition. The first inhibitor, EGTA, is a calcium chelator. In the case of cells stimulated to take up Ca^2+^, for example, through stimulation of P2X_7_ receptors with BzATP, EGTA may result in a decrease in intracellular Ca^2+^, thus preventing EMV release [22]. The second inhibitor, bisindolylmaleimide-I, is a protein kinase C (PKC) inhibitor preventing the externalisation of phosphatidylserine (PS) [23], which is a known mechanism that drives EMV release. The third inhibitor, imipramine, is an inhibitor of acid sphingomyelinase aSMase. Upon activation of the ATP receptor P2X_7_, MV shedding is associated with rapid activation and translocation of aSMase to the outer leaflet of the plasma membrane [24]. The fourth inhibitor, d-pantethine, blocks the translocation of phosphatidylserine [25], resulting in the non-externalisation of PS and therefore the inhibition of microvesiculation [26]. d-pantethine has been shown to reduce vesicle formation in mice infected with malaria compared to that of non-pantethine treated mice [27,28]. As it inhibits MV release, it so prevents systemic sclerosis in mice, as does knocking out the *ABCA1* gene [29]. The most effective inhibitor in this study, Cl-amidine, affects peptidylarginine deiminase activation, which causes post-translational protein deimination. We have previously described a novel pathway of MV biogenesis involving increased PAD-mediated protein deimination of cytoskeletal actin and nuclear translocation of PADs during microvesiculation [30,31]. Our current study confirms the effectivity of pharmacological PAD inhibition using Cl-amidine for significantly reduced cellular EMV release, targeting both MVs as well as demonstrating a novel inhibitory effect on exosome release. The sixth inhibitor, Y27632, is an inhibitor of Rho A. Rho-associated coiled-coil containing kinases (ROCK) are effectors of the small GTPase Rho-kinase, which amongst its myriad functions influences the redistribution of actin-cytoskeletal changes and regulates apoptosis-induced EMV release [32]. Inhibition by Y27632 prevents the activation of Rho A, which in turn abrogates EMV biogenesis [33]. The seventh inhibitor, calpeptin, is cell-permeable and inhibits calpain [34,35,36], even in the prostate cancer cell, PC3 [37], and reduces MV release from human embryonic kidney cells damaged with the streptococcal haemolytic exotoxin, streptolysin O [38]. The eighth inhibitor, glyburide, is another inhibitor of the ATP-binding cassette transporter and therefore a potential MV inhibitor [26], although in this study we found it to have no effect on EMV release. The ninth inhibitor tested, cytochalasin D, disrupts actin filaments of the cytoskeleton [39], specifically inhibiting actin polymerization, and it is suspected that, by modulating membrane trafficking [39], this may inhibit exosome release, as observed from HeLa cells [40]. Our data, however, indicated only a small reduction in exosome-sized vesicles and an increase in MV-sized vesicles. As expected, of the two endocytosis inhibitors used, both chlorpromazine, a cationic amphipathic drug, and MβCD, which depletes the cell plasma membrane of cholesterol [41], exclusively inhibited the release of exosome-sized vesicles.

In summary, Cl-amidine, and bisindolylmaleimide-I gave the highest inhibition of EMV release followed by imipramine and d-pantethine as detected by NanoSight analysis (Figure 1 and Figure 2A). However, d-pantethine significantly affected cell viability, which was found to be only 25% after 24 h (Figure 2B). Therefore, we chose to use Cl-amidine and bisindolylmaleimide-I, either individually or in combination, to inhibit EMV release in further experiments. Neither of these affected cell viability, indicating that inhibition of EMV release *per se* did not increase apoptosis.

Recently, we described a novel mechanism for PAD-mediated MV release. Specifically, this work showed that pan-PAD inhibitor Cl-amidine, inhibits MV release [37]. Here, we have further shown that, compared to other EMV inhibitors, Cl-amidine is very potent, inhibiting not only MV but also exosomal release (i.e., EMVs). Our data suggests that, rather than using sole EMV inhibitors, a combination of EMV inhibitors may be the way forward in enhancing the anti-neoplastic effect of pharmacological reagents. As demonstrated here in the PC3 prostate cancer and MCF-7 breast cancer cell lines, the percentage of viable cells was significantly lower when 5-FU treatment was carried out in the presence of Cl-amidine and the PKC inhibitor bisindolylmaleimide-I, compared to treatment with 5-FU alone, after only 24 h. While our results clearly indicate that we have also targeted inhibition of exosome release, this is currently based on size exclusion alone and will be followed up in our continuing studies using selective marker analysis.

Bisindolylmaleimide-I and Cl-amidine are likely to have multi-faceted effects on cells. As discussed by Pajak et al. [42], bisindolylmaleimide-I’s role as an anti-cancer therapeutic could include PKC-dependent and independent effects. These may include modulation of Wnt signalling, elimination of anti-apoptotic proteins, induction of intrinsic apoptosis, and reversal of MDR through restoration of sensitivity to chemotherapy, by inhibiting P-gp 1 (multidrug resistance protein 1- or ABCB1-) mediated drug efflux [42]. In the same vein, Cl-amidine will inhibit deimination of various known and unknown target proteins in the cell, including cytoskeletal actin and nuclear proteins such as histones via PAD nuclear translocation—which we have previously shown to be involved in MV biogenesis [37]. However, although in theory we cannot at present rule out possible off-target effects, neither EMV inhibitor on its own affected cell viability in the two cancer cell lines tested or at the concentrations used in this study.

Our previous work, using the calpain inhibitor calpeptin in the presence of 5-FU and docetaxel on prostate cancer cells, PC3, generated promising findings where combination therapy reduced the dose of docetaxel by 100-fold needed to produce comparable reduction in tumour volume in vivo [14]. We went on to show that methotrexate is released from cancer cells within MVs [14]. That chemotherapeutic drugs can be released within both exosomes and MVs has been well established by many other groups [12,13,14,15,16,17,18,19,20,21]. As our current work has shown several EMV inhibitors including Cl-amidine, bisindolylmaleimide-I and imipramine to be more potent even than calpeptin, this bodes well for future targeted combination therapy. Although all the inhibitors tested affected the number of EMVs released, methyl-β-cyclodextrin, inhibited exosome release only and Y27632 inhibited MV release alone. For future work, it would be interesting to assess the relative contribution of inhibiting either MV or exosome release on any sensitization of cancer cells to chemotherapy. It should be noted that as well as possibly influencing retention of chemotherapeutic drugs within cancer cells, EMVs may also transfer proteins involved in MDR such as P-gp [10]. Clearly therefore regulation and manipulation of EMV release is an important consideration to improving chemotherapy.

As the expanded repertoire of EMV inhibiting agents revealed in this study may also have the potential to sensitize cancer cells to chemotherapy, they must be further tested in vitro and in vivo, individually and in combination, taking advantage of their inhibitory effects on different EMV biosynthesis pathways. To increase the potency of chemotherapy further and effectively inhibit MDR, those EMV inhibitors that inhibit both exosomes and MVs will be of priority. Combination therapy involving selective EMV inhibitors may encourage re-testing of chemotherapeutic drugs currently not in favour due to severe side-effects and poor effectivity (such as 5-FU treatment of prostate cancer [43]). Additionally, looking ahead to the development of future in vivo experiments it is useful that no pre-treatment with the EMV-inhibiting drugs was needed to enhance the chemotherapeutic effect.

## 4. Materials and Methods

### 4.1. Maintaining Cell Lines

The adherent cell lines, PC3 and MCF-7 (ECACC), were maintained at 37 °C/5% CO_2_, in growth medium containing EMV-free Fetal Bovine Serum (Sigma-Aldrich, Dorset, UK) and RPMI (Sigma-Aldrich). Cells were split depending on confluence every 3–5 days. Cells were washed twice with EMV-free Dulbecco’s Phosphate Buffered Saline (DPBS; Thermo Fisher Scientific, Renfrew, UK), prepared as described before [37] and detached with 0.25% (*v*/*v*) trypsin/EDTA (Ethylenediaminetetraacetic acid; Sigma-Aldrich), which was removed after 10–15 min by centrifugation twice using EMV-free DPBS at 200× *g*/5 min (PC3 cells) or 120× *g*/5 min (MCF7 cells). Exponentially growing cells with viabilities of ≥95% were used in every experiment. The number of cells and viability were determined before all experiments using the viability assay, Guava ViaCount (Merck Millipore, Middlesex, UK).

### 4.2. Testing Capacity of Pharmacological Agents to Inhibit Cellular Release of EMVs

Cells were resuspended in pre-warmed serum-free RPMI and seeded in triplicate at 3.8 × 10^5^ cells/well in 12-well microtitre plates. EGTA (ethylene glycol-bis(β-aminoethyl ether)-*N*,*N*,*N*′,*N*′-tetraacetic acid; Sigma-Aldrich) (1.5 mM), bisindolylmaleimide-I (10 µM; Merck Millipore), imipramine (25 µM; Sigma-Aldrich), d-pantethine (1 mM; Sigma-Aldrich), Cl-amidine (50 µM; provided by P.R.Thompson), Y27632 (1 µM; Sigma-Aldrich), calpeptin (25 µM; Sigma-Aldrich), glyburide (10 µM; Sigma-Aldrich), MβCD (10 µM; Sigma-Aldrich), cytochalasin D (10 µM; Sigma-Aldrich), and chlorpromazine (10 µM; Sigma-Aldrich) were added separately and incubated for 24 h at 37 °C/5% CO_2_. EMVs released were isolated (4.4) and counted (4.5).

### 4.3. The Effect of EMV Inhibitors on 5-FU-Mediated Apoptosis of PC3 and MCF-7 Cells

Combinations of EMV inhibitors bisindolylmaleimide-I and Cl-amidine were used to test for a synergistic enhancement of the anti-cancer drug, 5-fluorouracil (5-FU; Sigma-Aldrich). PC3 and MCF-7 cells were maintained as before and seeded in triplicate at 3.8 × 10^5^ cells/well in 12-well microtitre plates with pre-warmed RPMI 1640 (Sigma-Aldrich). They were treated, either separately or in combination, with 10 µM bisindolylmaleimide-I and 50 µM Cl-amidine and at the same time with 1 µM 5-FU; control cells were treated with 1 µM 5-FU only. After 24 h, a cell death assay (Guava ViaCount, Merck Millipore) was performed using the Guava EasyCyte 8HT flow cytometer (Merck Millipore).

### 4.4. Isolation of EMVs

EMVs were isolated from the cell culture supernatant by centrifuging once at 200× *g*/5 min to remove cells. The supernatant was then centrifuged at 4000× *g*/60 min at 4 °C to remove cell debris and the new supernatant again at 25,000× *g*/1 h/4 °C. The isolated EMV pellet was resuspended in sterile-filtered (0.22 µm) EMV-free DPBS and centrifuged again at 25,000× *g*/1 h/4 °C to remove proteins possibly bound to the EMV surface (such as albumin). The EMV pellet was resuspended in sterile-filtered EMV-free DPBS and quantified by nanoparticle tracking analysis as described below (4.5). Isolated EMVs were used immediately.

The EMV pellet could then be centrifuged at 11,000× *g* for 30 min at 4 °C using a Beckman-Coulter Type 60 Ti rotor (Beckman Coulter Life Sciences, High Wycombe, UK) to separate microvesicles (MVs) and the resultant supernatant then centrifuged at 100,000× *g* for 1 h at 4 °C using the same rotor to isolate exosomes. Microvesicles and exosomes were characterised by flow cytometry for Annexin V-FITC (Abcam, Cambridge, UK) binding as a measure of phosphatidylserine exposition by transmission electron microscopy as described elsewhere [44] and by Western blotting for the exosome marker (CD63, Abcam) as previously described [31].

### 4.5. Nanoparticle Tracking Analysis (NTA, NanoSight LM10) of EMVs

Samples were diluted 1:50 using sterile-filtered, EMV-free DPBS, and the minimum concentration of samples was set as 5 × 10^7^ particles/mL. The settings at capturing stage were 8 and 13 for screen and camera gain respectively and at the process stage set at 9 and 3 for screen gain and detection threshold, respectively, as according to the manufacturer’s instructions (Malvern Instruments, Malvern, UK).

### 4.6. Flow Cytometry and Guava ViaCount Assay of EMVs

The Guava EasyCyte 8HT flow cytometer (Merck Millipore) and ViaCount assay (Merck Millipore) were used to count and determine viability of cells treated with EMV inhibitors, as previously described [45]. Briefly, the ViaCount assay distinguishes between viable and non-viable cells based on the differential permeability of two DNA-binding dyes in the Guava ViaCount reagent (Merck Millipore). The nuclear dye only stained nucleated cells, while the viability dye brightly stained necrotic or apoptotic cells.

### 4.7. Statistical Analysis

Statistical analysis was performed using GraphPad Prism version 6 (GraphPad Software, San Diego, CA, USA). Experiments were performed three times in triplicate. A one-way ANOVA was performed with Tukey’s post-hoc analysis. Differences were considered significant for *p* ≤ 0.05 (* *p* ≤ 0.05; ** *p* ≤ 0.01; *** *p* ≤ 0.001; **** *p* ≤ 0.0001).

## 5. Conclusions

This study emphasises the significance of EMV inhibitors in increasing the effectiveness of chemotherapy. Careful selection and combination of EMV inhibitors, targeting different EMV biogenesis pathways that influence exosome as well as MV release, can significantly enhance the effectiveness of anti-cancer drugs. The results presented in prostate and breast cancer cell lines encourage further testing in other types of cancer. Targeted prevention of possible EMV-mediated drug removal is likely to improve the efficacy of a range of drugs and thus poses a promising strategy to help overcome MDR.

## Abbreviations

BzATP	Benzoylbenzoyl adenosine triphosphate
Cl-amidine	Chloramidine
EMVs	Exosomes and microvesicles
5-FU	5-Fluorouracil
MDR	Multidrug resistance
MVs	Microvesicles
NTA	Nanoparticle tracking analysis
PAD	Peptidylarginine deiminase
